# An evaluation of Interprofessional group antenatal care: a prospective comparative study

**DOI:** 10.1186/s12884-017-1485-3

**Published:** 2017-09-07

**Authors:** Zoë G. Hodgson, Lee Saxell, Julian K. Christians

**Affiliations:** 1 South Community Birth Program, 202-1193 Kingsway, Vancouver, V5V 3C9 Canada; 2 Department of Biological Sciences Simon Fraser University, 8888 University Drive, Burnaby, V5A 1S6 Canada

**Keywords:** Connecting pregnancy, Group care, Interprofessional, Antenatal care, Perinatal outcomes, Client satisfaction

## Abstract

**Background:**

Maternal and neonatal outcomes are influenced by the nature of antenatal care. Standard pregnancy care is provided on an individual basis, with one-on-one appointments between a client and family doctor, midwife or obstetrician. A novel, group-based antenatal care delivery model was developed in the United States in the 1990s and is growing in popularity beyond the borders of the USA. The purpose of this study was to evaluate outcomes in clients receiving interprofessional group perinatal care versus interprofessional individual care in a Canadian setting.

**Methods:**

Clients attending the South Community Birth Program (SCBP), an interprofessional, collaborative, primary care maternity program, offering both individual and group care, were invited to participate in the study. Pregnancy knowledge and satisfaction scores, and perinatal outcomes were compared between those receiving group versus individual care. Chi-square tests, general linear models and logistic regression were used to compare the questionnaire scores and perinatal outcomes between cohorts.

**Results:**

Three hundred three clients participated in the study. Group care was comparable to individual care in terms of mode of birth, gestational age at birth, infant birth weight, breastfeeding rates, pregnancy knowledge, preparedness for labour and baby care, and client satisfaction. The rates of adverse perinatal outcomes were extremely low amongst SCBP clients, regardless of the type of care received (preterm birth rates ~5%). Breastfeeding rates were very high amongst all study participants (> 78% exclusive breastfeeding), as were measures of pregnancy knowledge and satisfaction.

**Conclusions:**

This is the first Canadian study to compare outcomes in clients receiving interprofessional group care versus individual care. Our observation that interprofessional group care outcomes and satisfaction were as good as interprofessional individual care has important implications for the antenatal care of clients and for addressing the projected maternity provider crisis facing Canada, particularly in small and rural communities. Further study of group-based care including not only client satisfaction, but also provider satisfaction, is needed. In addition, research into the role of interprofessional care in meeting the needs and improving perinatal outcomes of different populations is necessary.

## Background

Maternal and neonatal outcomes are influenced by the nature of antenatal care delivery [1]. Standard care during pregnancy is provided on an individual basis, with one-on-one appointments between a client and family doctor, midwife or obstetrician [2]. A novel, group-based maternity care delivery model was developed in the United States in the 1990s and is growing in popularity beyond the borders of the USA. The group model of care is based on the Centering Pregnancy concept, established in 1994 by midwife Sharon Schindler Rising [3]. Similar to standard care, the model involves assessment, support and education, bringing clients out of private clinic rooms and into groups for their care, with the added aim of building community and support amongst clients [3].

A number of previous studies have sought to evaluate Centering Pregnancy as a means of providing antenatal care [4–7]. In their recent review, Sheeder et al. [4] found group care to be associated with improved client and birth outcomes. Clients receiving group care participated more in their care and had higher satisfaction, increased breastfeeding rates and fewer preterm births than those receiving standard care. Benefits of group-based care have also been documented in terms of higher birth weights, especially for infants delivered preterm [5]. In their multi-site, randomized controlled trial, Ickovics et al. found group care to be associated with fewer preterm births, better maternal psychosocial function and satisfaction with care, and higher rates of breastfeeding, compared to standard care. Importantly, no differences in the costs of care delivery were found between group versus standard antenatal care [6]. Similarly, in their randomized controlled trial of antenatal care delivery models in a military setting, Kennedy et al. also found the group model of care to be as good as standard care in meeting clients’ needs [7].

Despite a number of studies having examined outcomes associated with group care [4–7], such studies have not spanned a wide range of demographics, and therefore may not be representative of other populations. Many have examined only particular demographics (i.e. certain races, ethnicities or ages, or specific elements of care, e.g., enjoyment), and few have included a comparison group. In addition, very few studies have been conducted in a Canadian context.

In Vancouver, British Columbia (BC), clients attending the South Community Birth Program (SCBP) are invited to join Connecting Pregnancy, a group-based antenatal care program based on the Centering Pregnancy model. Clients can alternatively opt to receive individual care on a one-on-one basis. Regardless of the model of care chosen, SCBP clients all receive interprofessional collaborative care, provided by midwives, family doctors, nurses and nurse practitioners. Every client is offered a doula at a subsidized rate. Forty-two doulas are part of the program, together offering 25 languages in addition to English. Doulas meet clients once prenatally, provide continuous support during latent and active phases of labour, and meet clients for a single postpartum visit.

A recent study compared perinatal outcomes in clients attending SCBP with those receiving standard care elsewhere. Outcomes in SCBP clients were compared with those of clients with a similar risk status who received standard care in community-based family physician, obstetrician and midwife practices. SCBP clients were less likely than these matched controls to undergo caesarean delivery and, among those with a previous caesarean delivery, more likely to plan a vaginal birth. Length of stay in hospital was shorter in the SCBP cohort for both the mothers and their newborns. SCBP clients were more likely than the matched controls to be breastfeeding exclusively at discharge [8]. However, this study did not permit us to discern which components of the interprofessional care program were responsible for the observed differences in outcomes between SCBP clients and the matched cohort.

The primary objective of the current study was to evaluate whether outcomes in SCBP clients differed between group versus individual prenatal care. Based on previous studies [4–7], we predicted that clients receiving group care would have improved knowledge, satisfaction, readiness for labour and birth scores, and better birth outcomes than clients receiving individual prenatal care. Group care not only provides standard antenatal clinical care but also presents additional opportunities for client learning (described below). Clients benefit from discussions, videos, and speakers and connect with other pregnant families, helping them to prepare for the course of pregnancy, labour and birth.

## Methods

Clients and partners attending the SCBP attend their first appointments with a family doctor or midwife on an individual basis. At 18–20 weeks, clients are invited to join Connecting Pregnancy group care with ten to eleven other clients and their partners whose estimated dates of delivery are in the same two to three week period. The groups are nine or ten 2-h sessions facilitated by a family doctor or midwife, alongside one of the SCBP nurses, with the same care provider and nurse for the majority of sessions. Each of the group sessions has a curriculum ranging from exercise and nutrition in pregnancy to preparation for labour, birth, breastfeeding and newborn care. In addition, the SCBP doula program coordinator attends three sessions to participate in the normal labour discussions and the role of doula support. Guest lecturers include a dietician, an exercise specialist, a physiotherapist and one of the SCBP doulas who teaches Tahitian dance. Initially, the Connecting Pregnancy groups are held once a month and then every two weeks as the pregnancy progresses. At each group session, the doctor or midwife spends a few minutes providing medical care for each client (the “belly check”). This involves reviewing any recent labs, abdominal palpation and listening to the fetal heart rate. If there are complications requiring consultation with an obstetrician or another specialist, or a personal issue requiring a one-on-one visit, this is booked outside the group schedule.

The majority of the clients entering group prenatal care are nulliparous clients. Occasionally, a multiparous client elects to join group care because their first baby was born a number of years ago or in a different country, or simply because they enjoyed the community they developed in the first group they attended. Clients not electing to join a group continue to have individual care provided by 2 or 3 midwives and/or family doctors. Women unable to speak and/or read English are not eligible for group care and receive individual care in the presence of a translator. The SCBP accepts low/medium risk clients into care. All clients with high risk pregnancies (e.g., cardiac disease, renal disease, preexisting insulin-dependent diabetes) or multiple gestations are transferred to an obstetrician before giving birth.

Between November 2012 and June 2014, clients in the first trimester of pregnancy attending the SCBP were invited to participate in the study at their first antenatal visit and given a consent form to consider. Clinic staff explained the study in detail and obtained written informed consent. All clients who completed at least one questionnaire (described below) were included in the study (no clients withdrew after completing a questionnaire). Women unable to complete the questionnaires in English were excluded from the study.

### Measures and outcome variables

Age, relationship status, ethnicity, number of previous births, mode of birth, preterm birth (less than 37 weeks gestation), gestational age at birth, low birth weight (less than 2500 g), newborn birth weight, admission to neonatal intensive care unit (NICU), and breastfeeding status at discharge (6 weeks postpartum) were recorded after reviewing each client’s chart.

Pregnancy knowledge was measured using a tool developed by Ickovics et al. to assess prenatal and infant care knowledge [6]. This questionnaire was administered in the first trimester and again in the third trimester. Readiness for labour and baby care were assessed in the third trimester only (at approximately 35 weeks gestation). The completion of the Edinburgh Postpartum Depression Scale (EPDS) questionnaire at 28–32 weeks gestation is part of standard clinical care in BC; despite being developed to detect postpartum depression, the EPDS has been validated for antenatal use [9]. Satisfaction with prenatal care was measured using an adaptation of the Patient Participation and Satisfaction Questionnaire [10] at discharge (6 weeks postpartum).

At the end of the study recruitment period, the detailed SCBP birth roster (an electronic clinical ledger with demographics of every client) was accessed to allow us to test whether our study participants represented a random sample of all SCBP clients (including those not part of the study), or whether they were a biased sample with respect to one or more of the following characteristics: maternal age, ethnicity, gestational age at birth, parity (nulliparous versus multiparous) and mode of birth.

### Statistical analyses

All statistical analyses were performed in SAS, Version 9.4. Chi-square tests, general linear models and logistic regression were used to compare Connecting Pregnancy group client characteristics, questionnaire scores and outcomes with those of clients receiving individual care. Part of the motivation for this study was to examine whether the availability of group care at SCBP contributed to differences in outcome between clients attending SCBP and those receiving standard care elsewhere [8]. Therefore, power analyses were conducted to assess the power to detect differences previously reported, i.e., a difference in the proportion of clients undergoing caesarean delivery (21.1% vs 31.3%) and in the proportion of clients breastfeeding exclusively at discharge (85.7% vs. 62.1%) [8]. Given the number of clients for which we had mode of delivery data (465 group and 374 individual), our power to detect previously-reported differences in rates of caesarean delivery in a two-sided test with a type I error rate of 0.05 was 0.92. We collected breastfeeding status for study participants only (207 group and 96 individual), and our power to detect previously-reported differences in rates of exclusive breastfeeding was 0.99.

To test whether our sample was representative of the population of clients accessing SCBP, we used a randomization approach, which involved (a) randomly selecting clients from among all SCBP clients to create a pseudosample of participants with the same sample size as the actual group of participants, (b) calculating the mean value of each trait of interest within the pseudosample, (c) repeating steps a and b many times to determine what the distribution of each trait looks like if participants are selected at random, and (d) comparing the values from the actual group of participants with the randomized distribution to determine whether the actual group is within the normal range of what is expected from random samples. The advantage of the randomization approach is that it is not subject to some of the assumptions of a traditional parametric t-test (e.g., normality). Clients receiving group care were analysed separately from clients receiving individual care (in case the bias was not the same in both care types), and the difference in bias between the two care types was tested.

## Results

### Study population

303 clients participated in the study. Of these, 96 chose individual care and 207 chose group care. Age was slightly higher in clients participating in individual care, and the proportion of nulliparous clients was much higher in group care than in individual care (Table 1). Caucasian clients were the largest ethnic group, and made up a higher proportion of clients in group care than in individual care (Table 1).

**Table 1:** Demographic characteristics of clients receiving group or individual care, among study participants and among all clients at South Community Birth Program (SCBP)

	Study participants		P (difference between group and individual)	All clients receiving care at SCBP		P (difference between group sample and all clients)	P (difference between individual sample and all clients)	P (difference between group and individual in sample vs. all clients)
	Group	Individual		Group	Individual			
Age (years)	32.4 ± 0.3	34.5 ± 0.4	0.0001^a^	33.1 ± 0.2	33.8 ± 0.2	0.0008	0.12	0.004
Parity								
Nulliparous	188/207 (91%)	25/96 (26%)	0.0001^b^	416/465 (89%)	112/374 (30%)	0.48	0.27	0.22
Multiparous	19/207 (9%)	71/96 (74%)		49/465 (11%)	262/374 (70%)			
Race								
Caucasian	132/207 (64%)	40/96 (42%)	0.0005^b^	272/465 (58%)	156/374 (42%)	0.03	0.91	0.29
East Asian	51/207 (25%)	44/96 (46%)		152/465 (33%)	147/374 (39%)	0.002	0.16	0.004
Other	24/207 (12%)	12/96 (13%)		41/465 (9%)	71/374 (19%)	0.08	0.08	0.01

Standard statistical tests were used to compare values between care types in study participants, and randomizations were used to determine whether study participants represented a random sample of all of clients at SCBP

^a^General linear model

^b^Chi-squared test

We performed randomizations to determine whether study participants represented a random sample of all of the clients receiving care at the clinic, including those not participating in the study, over the time period of the study (*N* = 374 in individual care, 465 in group care). Compared with all clients, study participants in group care were slightly younger, such that the difference between group care and individual care among study participants was significantly greater than that among the general SCBP population (Table 1). The proportions of nulliparous clients did not differ between study participants and all clients receiving care. The proportion of Caucasians was higher, and the proportion of East Asians was lower among clients participating in the study in group care compared to all clients in group care.

### Perinatal outcomes

Group care was associated with a lower rate of spontaneous vaginal delivery (SVD) and a higher rate of caesarean section than individual care (Table 2). However, there was a much higher proportion of nulliparous clients in group care (Table 1), and nulliparous clients would be expected to be at higher risk for caesarean section [11]. We therefore analysed mode of delivery by logistic regression including effects of care, parity, ethnicity, and maternal age. The effect of care type was not significant (Wald χ_1_^2^ = 1.9; *P* = 0.16), whereas the effect of parity was (Wald χ_1_^2^ = 5.3; *P* = 0.02), as was the effect of age (Wald χ_1_^2^ = 7.0; *P* = 0.01), with nulliparous and older clients being more likely to have some intervention. Similar results were obtained when analysing all SCBP clients (not only study participants), i.e., the effect of care type was not significant in logistic regression (Wald χ_1_^2^ = 0.23; *P* = 0.63). There were too few study participants to stratify analyses by parity (there were only 25 nulliparous study participants in individual care). However, among all nulliparous SCBP clients, there was no difference in mode of delivery between types of care (group: SVD 250/416 (60%), assisted vaginal delivery (AVD) 66/416 (16%), C/S 100/416 (24%); individual: SVD 66/112 (59%), AVD 20/112 (18%), C/S 26/112 (23%); Chi-squared test χ_2_^2^ = 0.26; *P* = 0.88).

**Table 2:** Mode of delivery in clients receiving group or individual care, among study participants and among all clients at South Community Birth Program (SCBP)

	Study participants		P (difference between group and individual)	All clients receiving care at SCBP		P (difference between group sample and all clients)	P (difference between individual sample and all clients)	P (difference between group and individual in sample vs. all clients)
	Group	Individual		Group	Individual			
SVD	111/207 (54%)	71/96 (74%)	Χ^2^_2_ = 11.5; *P* = 0.003	283/465 (61%)	271/374 (72%)	0.0026	0.62	0.06
AVD^a^	44/207 (21%)	10/96 (10%)		68/465 (15%)	30/374 (8%)	< 0.0001	0.22	0.15
C/S	52/207 (25%)	15/96 (16%)		114/465 (25%)	73/374 (20%)	0.85	0.34	0.28

A chi-squared test was used to compare values between care types in study participants, and randomizations were used to determine whether study participants represented a random sample of all of clients at SCBP

^a^AVD = Assisted Vaginal Delivery and includes both vacuum and forceps deliveries

Compared with all of the clients receiving care at the clinic over the time period of the study, including those not participating in the study, there was a significantly lower proportion of SVD, and a higher proportion of AVD, among study participants in group care than among all clients in group care. However, the difference in proportions between group and individual care did not differ between study participants and the general population. Furthermore, study participants represented a random sample of all clients with respect to caesarean section rates.

The rate of preterm birth was 5% in clients receiving both group and individual care, and similarly birth weight and EPDS score did not differ between group and individual care (Table 3). With regards to infant feeding, there were no differences between breastfeeding rates in clients receiving group versus individual care (Table 3).

**Table 3:** Other perinatal outcomes in study participants receiving group or individual care

	Group	Individual	P (effect of care)	P (effect of ethnicity)	P (effect of parity)	P (effect of age)
Preterm birth	11/207 (5%)	5/96 (5%)	0.79^a^			
Birthweight (g)	3362 ± 44	3360 ± 65	0.98^b^	0.003^c^	0.09	0.03^d^
EPDS	3.9 ± 0.3	3.8 ± 0.5	0.93^b^	0.04^e^	0.51	0.03^f^
Breastfeeding status						
Exclusive breastfeeding	173/206 (84%)	75/96 (78%)	0.37^a^			
Mixed feedings	26/206 (13%)	18/96 (19%)				
Formula	7/206 (3%)	3/96 (3%)				

^a^Chi-squared test

^b^General linear model including effects of care, ethnicity, parity and maternal age

^c^East Asians had significantly lighter babies than Caucasians

^d^Birth weight declined with increasing maternal age

^e^East Asians had significantly higher EPDS scores than Caucasians

^f^EPDS scores declined with increasing maternal age

### Knowledge, satisfaction and readiness

Group versus individual care did not affect knowledge at entry into care or in the third trimester or the change in knowledge between these two time points (Table 4). Care type also had no effect on readiness for labour, readiness for baby care, or satisfaction measured 6 weeks postpartum (Table 4).

**Table 4:** Knowledge, satisfaction and readiness scores in study participants receiving group or individual care

	Group	Individual	P for effect of care (group vs. individual)	P for effect of ethnicity	P for effect of parity	P for effect of age
	Sample sizes in parentheses					
Knowledge at entry into care ^a^	56.2 ± 0.5 (176)	55.2 ± 0.7 (88)	0.30	< 0.0001^d^	0.001^e^	0.06
Knowledge in third trimester^a^	59.5 ± 0.7 (94)	60.6 ± 1.0 (49)	0.41	0.01^d^	0.83	0.72
Change in knowledge	2.1 ± 0.8 (68)	3.9 ± 1.0 (42)	0.15	0.84	0.01^e^	0.09
Readiness for labour^b^	78.5 ± 1.9 (88)	78.4 ± 2.6 (47)	0.96	0.98	0.04^f^	0.19
Readiness for baby care^b^	80.1 ± 1.9 (90)	83.1 ± 2.6 (47)	0.40	0.63	< 0.0001^f^	0.46
Satisfaction^c^	114.7 ± 1.3 (89)	116.4 ± 1.8 (46)	0.51	0.11	0.51	0.25

Values are least squares means ± standard errors from a general linear model including effects of care, ethnicity, parity and maternal age

^a^Knowledge scale: maximum possible score = 72

^b^Readiness maximum possible score = 100

^c^Satisfaction maximum possible score = 125

^d^Higher among Caucasians

^e^Multiparous clients had higher knowledge at entry into care, whereas knowledge increased between entry into care and the third trimester more among nulliparous clients

^f^Multiparous clients had greater readiness for labour and baby care

## Discussion

In our setting, Connecting Pregnancy group care is comparable to individual care in terms of mode of birth, gestational age at birth, birth weight, breastfeeding rates, client satisfaction, pregnancy knowledge, and readiness for labour and baby care. The rates of adverse perinatal outcomes were extremely low regardless of the model of care chosen. Breastfeeding rates, measures of pregnancy knowledge and satisfaction were very high amongst all participants. These results are consistent with those of a recent Cochrane review including four studies from the USA, Sweden and Iran, which did not specifically focus on care by midwives and family doctors [2]. No differences in neonatal outcomes, perinatal mortality, spontaneous vaginal birth or breastfeeding initiation rates were found in clients receiving group versus standard individual care [2]. However, a number of previous studies have reported more favourable outcomes in clients receiving group care [4–6]. We speculate that there are two main reasons why these studies found a benefit of group care over individual care, whilst the current study did not. First, the nature of the individual care was unlike that in the current study and, second, the demographics differed substantially from our study population.

The BC midwifery model of practice incorporates the principles of continuity of care [12]. In BC and across Canada since the 1970s, the term “continuity of care” has been used to describe a relational model of care where clients get to know their providers through repeated visits with the same provider [12]. Whilst both midwives and family doctors provide care at SCBP, they all share a common philosophy and a consistent approach to practice, liaise regularly and meet frequently to coordinate care. A recent study found a cohort of SCBP clients to have lower caesarean section rates, shorter hospital stays and higher breastfeeding rates than clients receiving standard care outside of the SCBP, provided by an obstetrician, midwife or family physician [8]. This study did not permit an exploration of the elements of the birth program responsible for improved outcomes. However, the current study suggests that elements common to both individual and group care are associated with these favourable outcomes. Although the caesarean delivery rate was high (25%) in group care in our study, 91% of these clients were nulliparous and so at higher risk of intervention. This caesarean delivery rate is very similar to that previously reported for nulliparous women (24.1%) at SCBP, including both group and individual care clients [8]. This rate is also substantially lower than the provincial average (~30% in 2009–2010) [11], and SCBP clients have previously been found to have significantly lower caesarean rates than matched controls receiving standard care [8]. Thus, while much work remains to be done, the models of care at SCBP, whether group or individual, are a step towards lowering caesarean rates among first births.

A Cochrane review found outcomes amongst clients who had midwife-led models of care to be more favourable than other models of care [13]. Boss et al. found that continuity in prenatal care resulted in less neonatal morbidity, higher birth weights, more appropriate maternal weight gain and higher Apgar scores at one and five minutes [14]. In the Ickovics studies [5, 6], group care may have had greater continuity of care than standard individual care, which was provided by an obstetrician or a midwife (Ickovics, *pers. comm.* 2016). In contrast, in the current study, individual and group care outcomes were equivalent due to the preservation of continuity in both models.

Care providers at the SCBP respect the right of clients to make informed choices and facilitate this process by ensuring there is adequate time for discussion during antenatal appointments [12]. Normally, individual antenatal and postnatal visits last between 30 to 45 min. This is in contrast to standard obstetrical care where appointments are usually 10–15 min long. In the studies where outcomes were more favourable in clients receiving group care, the authors have suggested that the benefits stem from the content and intensiveness of prenatal care received in the group context. The extended time spent together during group prenatal visits is thought to afford a greater understanding of healthy behaviours during pregnancy, the acquisition of more prenatal knowledge and, in turn, the adoption of health-promoting rather than health-damaging behaviours [6]. The educational and birth preparedness component of antenatal appointments in *both* individual and group care at the SCBP may explain the lack of differences in outcomes in this study.

The demographics of our study population may also explain why outcomes associated with group care were not found to be superior to those in clients receiving individual care. Eighty percent of participants in the Ickovics study identified as African American [6], whereas the largest ethnic group in the present study was Caucasian. This demographic difference may explain the difference between rates of preterm birth. Black clients are at higher risk of preterm birth than Caucasian clients in Canada and the USA [15, 16]. As a result, the study of Ickovics had higher rates of preterm births, which may have increased the power to detect an effect of care delivery model. In addition, the mean age of participants in the Ickovics study was 20.4 years compared to early to mid-thirties in the current study. The younger population may have entered the Ickovics study with less knowledge of pregnancy due to having fewer peers who had given birth. A greater extent of knowledge may therefore have been acquired in the group setting than in the individual setting. However, there is a paucity of research examining how pregnancy-related knowledge varies with maternal age, aside from studies that have compared pregnancy knowledge in clients of advanced maternal age to a younger cohort (e.g., see [17]).

Further differences in the populations may have explained the beneficial effects of group care in the Ickovics study. The study participants in the Ickovics study were recruited from clinics that primarily serve minority clients of lower socioeconomic status (SES). It is well known that SES is one of the most reliable predictors of health disparities [18]. A high rate of risk-taking behaviours, such as smoking and alcohol use have been documented in low-SES individuals [19], which increase the risk of adverse perinatal outcomes such as low birth weight infants and preterm birth [18]. It is perhaps due to the lower SES of the individuals in the Ickovics study that group care had more power to exert a positive influence on outcome. Indeed, Palmer, Cook and Courtot found low-income clients benefitted more than most from the allocation of additional and nontraditional maternity care resources such as prenatal group care [20]. Palmer et al. believe that such components of care help providers address underlying social risk factors that may be negatively affecting the health of the clients and their unborn children [20]. While we did not measure SES directly in our study, SCBP does not primarily serve lower SES communities.

A limitation of the present study is that it was not randomized so differences in the nature of the clients choosing the different models of care cannot be eliminated.

## Conclusions

This is the first Canadian study to compare outcomes in clients receiving group versus individual antenatal care. Perinatal outcomes and measures of client knowledge, satisfaction and labour and birth preparedness were comparable between clients regardless of model of care received. Our observation that interprofessional group care is as effective as interprofessional individual care has important implications for the antenatal and postnatal care of clients, as well as the potential of addressing the projected maternity care provider crisis facing Canada [21]. Further study of group-based care including not only client satisfaction but also provider satisfaction, as well as the long-term benefits of group care, is also needed. In addition, research into the role of interprofessional care in meeting the needs and improving perinatal outcomes of different populations is necessary.

## Abbreviations

AVD: Assisted Vaginal Delivery (includes vacuum and forceps)
BC: British Columbia
C/S: Caesarean Section
EPDS: Edinburgh Postpartum Depression Scale
NICU: Neonatal Intensive Care Unit
SCBP: South Community Birth Program
SES: Socioeconomic Status
SVD: Spontaneous Vaginal Delivery
